# Physical Activity Counseling in Primary Care in Germany—An Integrative Review

**DOI:** 10.3390/ijerph17155625

**Published:** 2020-08-04

**Authors:** Eszter Füzéki, Theresa Weber, David A. Groneberg, Winfried Banzer

**Affiliations:** Division of Preventive and Sports Medicine, Institute of Occupational, Social and Environmental Medicine, Goethe-University Frankfurt, 60590 Frankfurt, Germany; t.weber@med.uni-frankfurt.de (T.W.); groneberg@med.uni-frankfurt.de (D.A.G.); banzer@med.uni-frankfurt.de (W.B.)

**Keywords:** physical activity counseling, health care, exercise on prescription

## Abstract

Physical activity counseling in primary health care is regarded as a useful complementary preventive and therapeutic measure and is advocated by leading public health institutions. This integrative review summarizes the available data on physical activity counseling in primary care in Germany. A systematic literature search in various databases (peer reviewed and grey literature) was carried out for quantitative and qualitative studies on physical activity counseling and use of “Exercise on Prescription”. The 25 studies included show a very high methodological diversity and, in some cases, considerable risks of bias, with limited comparability across studies. Counseling was provided in all studies by physicians. They report frequent physical activity counseling, which is partly confirmed and partly refuted by patient data. The use of “Exercise on Prescription” is at a very low level. Information on the frequency of physical activity counseling in Germany varies depending on data source and is sometimes contradictory. Our review provides a synthesis of various perspectives on routine physical activity counseling in primary care in Germany. Future studies using standardized and validated instruments in representative samples are needed to further knowledge on counseling and to be able to establish trends in prevalence. Strengthening the topics of physical activity and health and physical activity counseling in medical curriculum is strongly recommended.

## 1. Introduction

The evidence on the wide-ranging health benefits of regular physical activity (PA) is overwhelming [1,2]. PA reduces mortality risk, the risk of chronic diseases with the highest disease burden, such as cardiovascular and metabolic diseases, cancers, and diseases of the musculoskeletal system, and is also an effective (complementary) therapeutic measure for these clinical conditions [2]. Nevertheless, PA levels remain low worldwide [3] and in Germany [4].

The relevance attributed to routine PA promotion in primary care is based on two further aspects in addition to the health effects of PA. Through universal access to health care in most Western countries, physicians can reach practically all social-economic groups, and physicians are considered the most important source of health information. Because of this high public health potential, PA counseling in health care has been advocated by a number of public health institutions, including the World Health Organization [5]. In Germany, the Annual Meeting of German Physicians has also recently confirmed the importance of PA counseling as a part of physicians’ routine [6].

In international practice, two general approaches in PA promotion in health care are established: PA counseling, where counseling is provided by physicians and/or other health care professionals and patients implement the recommendations on their own; and exercise referral (also called exercise on prescription, green prescription), where physicians refer patients to an existing group offer, usually in a community setting. An increasing number of countries worldwide have established exercise referral schemes and developed PA counseling programs [7,8].

Exercise referral schemes [9] and PA counseling [10,11] have been shown to increase participants’ PA levels at least at short or middle term, and PA promotion interventions in primary care can yield clinically relevant effects [12].

Little is known about the current level of routine PA promotion in primary care in Germany. The main aim of this study is to provide an overview in the form of an integrative review [13] of the prevalence of PA counseling in primary care and the use of the German Exercise on Prescription (EoP) program. Further, we aim to summarize data on the content and effects of, as well as barriers to routine PA counseling.

## 2. Materials and Methods

The following study was prepared according to the Preferred Reporting Items for Systematic Reviews and Meta-Analyses (PRISMA) guidelines [14]. The systematic literature search, data extraction, and the assessment of the risk of bias in the individual studies were performed independently by two researchers (E.F., T.W.). Differences in opinion relating to inclusion and exclusion criteria were discussed until consensus was reached.

The literature search was performed in the following databases: PubMed, Web of Science, Google Scholar (first 10 pages), Karlsruher Virtueller Katalog (database for diploma, master, state examination, bachelor, and master theses), diplom. de, dissonline, base-net Bielefeld Academic Search Engine, DART-Europe E-theses Portal, Open Access Theses and Dissertations, as well as in relevant the German language journals not listed in PubMed (Bewegungstherapie und Gesundheitssport, Deutsche Zeitschrift für Sportmedizin, Prävention und Gesundheitsförderung, Public Health Forum, Journal of Public Health, Der Kardiologe, MMW—Fortschritte der Medizin, Der Internist, Der Orthopäde, German Journal of Exercise and Sport Research—Sportwissenschaft, Zeitschrift für Allgemeinmedizin) for the period 2000–2019 in German and English using the search terms Bewegungsberatung, Rezept für Bewegung, physical activity counseling AND Germany, exercise prescription AND Germany (search terms linked with AND were considered together). In addition, the reference lists of the included sources were searched, and a forward reference search was performed.

The following a priori inclusion criteria were defined: (1) studies on prevalence of routine PA counseling or use of Exercise on Prescription in primary care in Germany, (2) publication language English or German, (3) quantitative or qualitative studies, (4) peer reviewed and not peer reviewed (grey) literature. We excluded studies on short-term PA counseling interventions (i.e., non-routine PA counseling) and studies in which PA counseling did not take place in primary care, as well as studies on preventive counseling services in which the share of PA counseling could not be determined. Data extracted from the included studies are summarized in Table 1 and Table 2.

The risk of bias was assessed using the 10-item instrument developed by Hoy and colleagues [40] for quantitative studies. The instrument addresses four domains of bias and provides a summary risk-of-bias assessment. The overall interrater agreement is 91% with a Kappa statistic of 0.82 [40]. Risk of bias in qualitative studies was assessed using the 10-item Critical Appraisal Skills Programme (CASP) checklist [41].

## 3. Results

**Figure 1:**
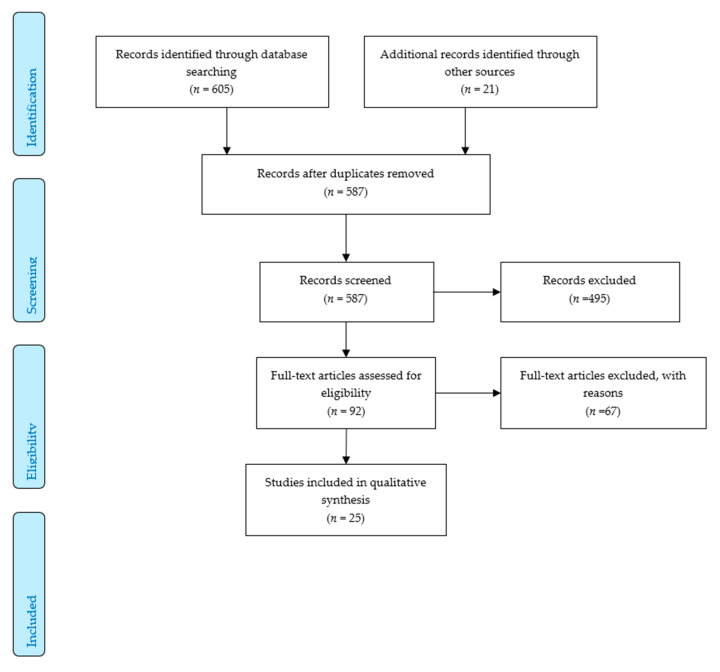
Flow chart.

The search yielded 626 records. After deduplication, we screened 587 titles and abstracts and reviewed 92 full texts subsequently. After applying the inclusion and exclusion criteria, 25 articles from 20 studies were included in the descriptive analysis [15,16,17,18,19,20,21,22,23,24,25,26,27,28,29,30,31,32,33,34,35,36,37,38,39], cf. also Figure 1.

Nineteen studies were quantitative, eight of which were conducted with patients, ten with physicians, and one study was based on patient records. Of the six qualitative studies, three were conducted with physicians and one with patients. In two studies, physician–patient discussions formed the data basis, cf. also Table 1 and Table 2. Four studies are grey literature [33,37,38,39].

PA counseling [17,23,24] and the use of EoP per se [18,25,39] were primary research questions in three studies each. The remaining publications represent secondary research questions of other, usually more comprehensive studies, such as cardiovascular disease prevention in primary care [26] or the National Health Survey [15,16,20].

Due to the great methodological diversity of the included studies, a meta-analysis was not feasible.

### 3.1. Study Quality

The results of the methodological assessment are presented in Table 3 and Table 4. 

No study has given a formal definition of “physical activity” or “physical activity counseling”; various terms and periphrases were used instead. All quantitative studies that did not evaluate data in patient records used self-developed survey instruments (questionnaires), with one or more items for PA counseling or use of EoP. Physicians were typically invited to provide information on the prevalence of counseling using different level Likert scales. The overall sample of patients to whom the counseling prevalence refers varied and was not explicitly mentioned in every study. None of the physician surveys provided information on how inactive or insufficiently active patients were defined and identified. Patient surveys included questions on PA counseling and prescribing exercise in different past time-periods.

### 3.2. Content of PA Counseling

Beyond data on prevalence, some studies provide information on the content and methods of counseling, such as recommendations for specific types of PA [23,37]; general information on the health benefits of PA [18,23]; recommendation on the frequency and intensity of PA (Kroll 2014); patients’ preferences [35]; and disease-related, individual exercise capacity [23]; use of written materials [24,29]; referral to group offers or to therapists [17,24,29,32]; written agreement on goals and follow-up [27,29], and motivational counseling [29].

### 3.3. Self-Assessed Competences and Knowledge, Ability to Motivate

Two studies [26,29] and [24] have assessed physicians’ self-rated counseling competence and knowledge. The physicians report high to very high competences and at the same time express doubts that they can actually bring about behavior change in patients [23,24,26,29]. Similar views are also voiced in qualitative studies [34,39].

### 3.4. Barriers

Some studies have assessed barriers to routine PA counseling [23,24,25,39]. These included lack of remuneration, lack of time, patients’ disinterest and lack of compliance, lack of information, and lack of networking with partners outside the health care system [17,25,39].

### 3.5. Effects of Counseling

The effects of counseling or prescription of PA were assessed in three studies using non-validated self-reports with different follow-up periods [17,18,20]. No study has used objective measurement methods. Kroll documented the effects of counseling in her qualitative study [37].

## 4. Discussion

The first aim of this review was to present data on the prevalence of routine PA promotion in health care in Germany as comprehensively as possible. Our approach was that of an integrative review to “enhance a holistic understanding” of this topic [13]. The second aim was to offer and discuss findings on contents of and barriers to PA counseling. The great methodological diversity, which is inherent in the method of integrative reviews, and the substantial methodological limitations of the studies included make it difficult to draw a conclusive summary. Since to date no review on PA counseling in primary care in Germany has been published, we adopted an approach that allows for the synthesis of different perspectives on the topic. Thus, e.g., the juxtaposition of contrasting physician and patient reports adds a further dimension relative to presenting just “one side” [13].

### 4.1. Prevalence of Counseling

Physician-reported prevalence of counseling is high. The largest nationwide study, with over 4000 respondents, found that 71.8% of primary care physicians offered PA counseling to more than half of their patients [26]. Furthermore, more than 80% of neurologists surveyed in a nationwide study stated that they “frequently” counseled their patients on PA [23]. Moreover, 90% of the general practitioners surveyed in Berlin report offering PA counseling always or frequently if it is indicated [31]. General practitioners in and around the city of Würzburg also give recommendations on PA physical activity to 53.5% of older patients [24]. However, knowledge and use of EoP is limited: less than 8% of the physicians surveyed use it as part of their PA counseling [25] or do not use it in the intended sense [39].

Some, but not all, of the patient-reported data seem to contradict those of the physicians. The representative data of the National Health Surveys show a considerably lower prevalence: 8.6% of patients between 18 and 64 years of age report having received PA counseling in the past 12 months [16]. According to the 1998 National Health Survey, the prevalence of counseling in the 18–79 age group was as low as 6.85% [20]. However, two smaller studies documented an almost fourfold (32.8%) [19] and sevenfold (48%) [17] prevalence of counseling, respectively, in older patients. In a sub-sample of the Leipzig Life Study, 21.5% of patients reported having received PA counseling from their primary care physician [22].

The only study based on patient records found a counseling prevalence of 21.4% [33].

Counseling prevalence seems to be higher in patients with diabetes [16,19] coronary heart disease [16,19], myocardial infarction, osteoarthritis, multi-medication [19], and hypertension [16] than in people without these conditions. These patient-reported data are consistent with those of physicians: physicians with a high proportion of high-risk patients seem to offer counseling more frequently [29]. These results are also in line with data from Sweden [42], the U.S. [43,44], and a systematic review [45].

### 4.2. Contents of Counseling

Current data from Germany provide little insight into how PA counseling is offered. It remains largely unknown whether counseling is based on a theory of behavior change, whether physicians use motivational techniques and, if so, which ones, how they define “inactivity”, for which patients they consider counseling to be indicated, how often follow-ups take place. These data would be of major interest when it comes to effectiveness, since though the specific intervention components associated with best result cannot be clearly defined, interventions that include multiple behavioral change strategies such as goal setting, written prescriptions, providing feedback, and follow up, seem to yield better outcomes [12].

### 4.3. Barriers

Primary care physicians’ attitudes and perceptions on PA counseling is very similar to those reported from other countries [45]. Physicians typically regard lifestyle counseling in general [26] and PA counseling in particular [25,39] as an important part of their routine as medical professionals, but face a number of barriers. Besides lack of time [23,24,39], patient-related factors such as disinterest, lack of motivation, and lack of compliance [23,24,25,39] are often reported to be important barriers to routine counseling.

There seems to be a disconnect between physicians’ and patients’ perception of success in behavior change, which is very similar across countries. While physicians in Germany [23,24,25,34,39] and elsewhere [45] cite patients’ disinterest and reluctance to act upon advice as one of the major barriers to counseling, patients’ reports seem to at least to some extent contradict these relatively widespread assumptions. Indeed, several German studies show that patients value physicians’ advice. More than three-quarters of older patients stated that they had decided to keep up with an exercise course recommended by their family doctor, and 82% were generally more interested in a course if their family doctor recommended it [17]. More than half of the patients who received an EoP from their physician reported that they did more exercise and were more active in their everyday life [18]. In the National Health Survey, compliance rate upon counseling was 52% [20]. Appreciation of physicians’ support in increasing PA has been found in various countries and patient groups [46,47,48].

Lack of remuneration for counseling is mentioned in every study that identified the barriers [23,24,25,26,29], but interestingly, it is not always considered the most important factor.

### 4.4. Findings in Relation to Other Countries

The widespread call and advocacy for routine PA promotion in primary care notwithstanding there seems to be a paucity of current representative data on PA counseling prevalence. Representative patient-reported data indicate that in 2010 about one third of all U.S. patients who had seen a physician or other health professional in the previous 12 months had received advice on PA [43]. In a national sample, which was representative in some but not all relevant terms, 18.2% Australian adults reported having received PA counseling from their physician in the previous 12 months [49].

In a nationwide Brazilian study, over 80% of physicians reported regularly providing PA counseling [50]. A nationally representative survey of primary care physicians in the United States found that 93.9% and 86% provide guidance on PA “often” or ”always” to patients with and without chronic diseases respectively [44]. In a national survey among Canadian primary care physicians, 85% of respondents reported asking their patients about PA, whereas only 15.8% provided written advice [51]. Similar rates have been reported from Ireland [52]; 88% of survey participants reported asking about PA, but the vast majority (82.6%) did not provide written prescription [52]. These findings collectively suggest considerably higher physician-reported prevalences than patient-reported ones.

Based on electronic patient records, an EoP was issued to 3% of all patients in primary and secondary care in a Swedish County Council [42].

Involving allied health care professionals, such as nurses, physiotherapists, or exercise scientists, into PA counseling in primary care is practice in some countries [53]. This interdisciplinary model has been shown to produce better result than physician-only approaches [53]. We could identify no study in Germany where professions other than physicians were involved. The less than optimal cooperation between professions and sectors was cited as a barrier in various studies [17,18,25,34,39]. We see improved interdisciplinary work as a key element to enhance the prevalence of PA counseling in primary care.

Direct comparison between countries is challenging for various reasons. Assessment methods (self-report vs. patients’ records), data sources (patients vs. physicians), patient and physician characteristics differ in different countries. Interestingly, data showing that physicians tend to offer advice on PA more readily to already diseased populations than to currently healthy participants seems to be consistent across countries, data sources, and assessment methods [16,42,45,49]. Encouraging patients with chronic diseases and compromised health to be more physically active is very welcome. On the flipside, PA counseling seems to be underutilized as a preventive tool.

### 4.5. Strengths and Limitations

To the best of our knowledge, this is the first study to give an overview of PA counseling in primary care in Germany. We have followed the strict criteria of the PRISMA recommendations. In order to provide the most comprehensive overview possible, we have included both quantitative and qualitative studies from peer reviewed and grey literature. At the same time, our review must be seen in the light of the limitations of the studies included.

There are no widely accepted reporting schemes for survey studies, which leads to inconsistent reporting [54]. In the included studies, with a few exceptions, response rates were low, and most studies did not provide information on item non-response (complete vs. partial answers to the questions). We cannot exclude the possibility that the data presented here contain a positive bias. Self-selectivity may have played a role for both physicians and patients, and physicians may have indicated more frequent counseling activity (social desirability). Overall, the methodological limitations greatly reduce the generalizability of the results.

## 5. Conclusions

Data on the prevalence of PA counseling in Germany vary according to data source and are sometimes contradictory. Direct comparison with other countries is challenging due to methodological issues. Perceived barriers to routine PA counseling in primary care seem to be very similar to those reported from other countries. To improve comparability among studies and to improve overall methodological quality, standardized instruments should be developed and validated. Surveys in representative samples using such instruments are needed to further knowledge on counseling and to be able to establish prevalence trends. Conducting studies on counseling methods and contents can add valuable information beyond prevalence. Strengthening the topics of physical activity and health and physical activity counseling in medical curriculum is strongly recommended.

## Figures and Tables

**Table 1:** Quantitative studies.

Patients Surveys									
Study	Primary Research Question	Secondary Research Question	Survey Instrument and Survey Mode	Sample	Place and Time of the Survey	Response Rate	Statistics, Dealing with Missing Values	Main Results	Risk of Bias According to Hoy et al.
[15]	Representative data on health of the general population. Health services utilization	Prevalence of physicians’ PA ^1^ counseling in the 12 previous months; time trends and regional differences	Self-developed 1-item instrument Validity n.r. ^2^ Paper-pencil survey. Self-report	BGS 98 ^3^ und DEGS 1 ^4^; representative sample *n* = 11,907 Between 18 and 64 years	Nationwide; 1997–1999 and 2008–2011	n.r.	Logistic regression; missing values: n.r.	Prevalence of physicians’ PA counseling dropped significantly from 10.1% (BGS 98) to 8.6% (DEGS 1) (OR ^5^ 0.83, 95% CI ^6^ 0.72–096) Higher prevalence in larger cities than in rural regions (BGS 98: OR 1.8, 95% CI 1.26–2.58; DEGS 1: 1.49, 95% CI 1.01–2.20)	5/10
[16]	Representative data on health of the general population. Health services utilization	Prevalence of physicians’ PA counseling in the 12 previous months. Participation in preventive PA courses	Self-developed 1-item instrument Validity n.r. Paper-pencil survey. Self-report	BGS 98 and DEGS 1; representative sample *n* = 11,907 Between 18 and 64 years	Nationwide; 1997–1999 and 2008–2011	n.r.	Logistic regression; missing values: n.r.	Prevalence of physicians’ PA counseling dropped between 1997–1999 and 2008–2011, increased prevalence of physicians’ PA counseling in diabetics (OR 3.42, 95 % CI 1.68–6.96) and patients with cardiometabolic risk factors (OR 5.33, 95 % CI 1.89–15.00) Individuals who receive counseling are more likely to participate in preventive PA courses	5/10
[17]	Relevance and role of general practitioners in increasing PA in elderly patients	Exercise	Self-developed instrument with four thematic blocks pretest Validity n.r. Paper-pencil survey in waiting room. Self-report	*n* = 400 ≥70 years	25 practices in the Federal State of Rhineland-Palatinate; November 2018–April 2019	324/400 (81%)	Descriptive analysis; missing values: n.r.	48% have received counseling at least once; 52% report to be “more or less active”; 52% would like to become more active, of which 93% has no information on suitable courses; 88% would welcome in physicians cooperated more with health oriented PA providers	4/10
[18]	Perceived quality of counseling on Exercise on Prescription. Intention and consequences following a counseling on Exercise on Prescription		Self-developed 17-item instrument Validity n.r. Paper-pencil survey. Self-report	Patients who have received counseling on Exercise on Prescription *n* = 173	12 Practices in 8 “Sports Regions” in the Federal State of Hessia January–March 2014	51/173 (29.48%)	Contingency table, OR, missing values: n.r.	Counseling mainly perceived as positive, increased awareness for PA and health, 53% report doing more exercise, 51% more active in daily life	4/10
[19]	Prevalence of peripheral arterial disease in the elderly in family practice	Prevalence of family practitioners’ PA counseling in the 12 previous months in the elderly	Self-developed 1 item instrument in the getABI Study ^7^ Computer-assisted telephone interview Validity n.r.	Participants of the getABI Study *n* = 5578, ≥65 years	Family practitioners nationwide 2008	1937/5578 (7 years follow-up) 193/1627 (29.16%)	Logistic regression missing values: n.r.	32.8% of patients report having received PA counseling men (OR 1.34, 95% CI 1.06–1.70). patients with pain (OR 1.43, 95% CI 1.13–1.81). with coronary heart disease and/or myocardial infarction (OR 1.56, 95% CI 1.21–2.01). Diabetes mellitus (OR 1.79, 95% CI 1.39–2.30) and arthritis (OR 1.37, 95% CI 1.08–1.73). and patients with multi-medication (>5 medications (OR 1.41, 95% CI 1.11–1.80)	5/10
[20]	Representative data on health of the general population. Health services utilization	Compliance following physicians PA counseling; prevalence of physicians’ PA counseling in the 12 previous months	Self-developed 2-item instrument Validity n.r. Paper-pencil survey. Self-report	BGS 98 representative sample *n* = 7124 Between 18 and 79 years	Nationwide; October 1997–March 1999	n.r.	Chi-square test, t-test, logistic regression missing values: n.r.	Prevalence of physicians’ PA counseling: 6.85%, in patients ≥70: 0% Compliance: ca. 50%. Compliance higher in women, non-smokers, and healthy eaters	5/10
[21]	Attitude, perceived need of counseling, counseling received in family practice patients	Mentioning PA and health	Self-developed instrument, Tested and validated in a pilot study Validity n.r.	EUROPREVIEW-Study ^8^ *n* = 370 between 30 and 70 years	In and around the City of Cologne September 2008–September 2009	Rate of consenting practices 66%. Rate of participation among patients 70%	2-sided Chi- square test missing values: n.r.	PA and health mentioned in the previous 12 months: 39.4%. PA and health ever mentioned: 54.7% 31% patients would welcome more support and counseling on PA (vs. 57% in Europe)	3/10
[22]	Population study on chronic diseases	Prevalence of physicians’ PA counseling (sub-sample)	Self-developed instrument Validity n.r.	Life-Adult Study; *n* = 2244 between 19 and 79 years	Leipzig March 2012–May 2013	1171/2244 patients received health counseling, of those 482 received PA counseling	Chi-square test missing values: n.r.	21.5% of all patients have received PA counseling	3/10
**Physician Surveys**									
**Study**	**Primary Research Question**	**Secondary Research Question**	**Survey Instrument and Survey Mode**	**Sample**	**Place and Time of the Survey**	**Response Rate**	**Statistics, Dealing Missing Values**	**Main Results**	**Risk of Bias According to Hoy et al.**
[23]	PA counseling by neurologists	Facilitators and barriers	Self-developed instrument with closed and open questions Validity n.r. Online survey	Members of the German Neurologist Association	Nationwide September 2015	169/784 (21.6%)	Cramer’s Index, Contingency table, OR, missing values n.r.	Prevalence of physicians’ PA counseling: 80.5% often, 13% occasionally, 77.5% provide general information, 66.9% detailed information regarding specific exercise forms. 82.2% consider individual and disease-specific circumstances, 69.2% would provide PA counseling more often and more in depth. Barrier: patients’ disinterest, physically active physicians provide counseling more often	5/10
[24]	PA counseling by family physicians for elderly patients Barriers to knowledge and skills Need and interest in training Perceived quality of a training		Self-developed 42 and 32 item instruments respectively Validity n.r. paper-pencil survey (per mail and in person) self-report	Family physicians *n* = 60 *n* = 22	City of Würzburg and vicinity June–September 2015	60/291 20.62% (Counseling) 22/23 95% (Training)	Descriptive analysis	Approx. 50% of patients receive counseling Barriers: Lack of time, patients’ disinterest Physicians highly interested in training on PA counseling	5/10
[25]	physicians’ knowledge and use of Exercise on Prescription, barriers to use		Self-developed instrument Validity n.r. Pre-test paper-pencil survey per mail Self-report	All general practitioners in two districts of Eastern Bavaria *n* = 2821	Oberpfalz and Nieder-Bayern June–November 2013	923/2821 (32.7%)	Descriptive analysis	26.4% know Exercise on Prescription, 70.1% of those do not use it Barriers: lack of information on Exercise on Prescription, local offers, lack of reimbursement of costs of courses	8/10
[26]	Family physicians’ attitude to lifestyle counseling Barriers to lifestyle counseling	PA counseling. Significance of PA, skills and techniques to motivate patients	Self-developed instrument Validated via cognitive interviews Pre-tested in pilot study Validity n.r. Paper-pencil survey per mail and online survey Self-report Compensation of €20 for participation	ÄSP-kardio-Study ^9^ Representative sample of German family physicians, a priori defined sample of 13,294	Nationwide; October 2011–March 2012	4074/13,294 (RR3 33.9%)	Descriptive analysis	71.8% routinely provide PA counseling (i.e., to more than 50% of the patients) 100% of physicians judge PA to be important. 87% report good or very good knowledge, 48% report being successful in PA counseling	8/10
[27]	Regional differences in physicians’ (1) attitudes to lifestyle counseling, (2) lifestyle counseling, (3) perceived barriers to lifestyle counseling	Assessment of PA, PA counseling, monitoring	Self-developed instrumentValidated via cognitive interviews Pre-tested in pilot study Validity n.r. Paper-pencil survey per mail and online survey Self-report Compensation of €20 for participation	ÄSP-kardio-Study Representative sample of German family physicians, a priori defined sample of 13,294	Nationwide; October 2011–March 2012	4074/13,294 (RR3 33.9%)	Chi-square test. Kruskal–Wallis test. Logistic regressions, missing values n.r.	Physicians in practices in rural regions provide assessment of PA, PA counseling, and monitoring less frequently than physicians in urban areas	8/10
[28]	Gender differences in lifestyle counseling	PA counseling	Self-developed instrument Validated via cognitive interviews Pre-tested in pilot study Validity n.r. Paper-pencil survey per mail and online survey Self-report Compensation of €20 for participation	ÄSP-kardio-Study Representative sample of German family physicians, a priori defined sample of 13,294	Nationwide; October 2011–March 2012	4074/13,294 (RR3 33.9%)	Chi-square test. Mann–Whitney U test. Logistic regressions, missing values n.r.	Female physicians assess PA more often (OR 1.39)	8/10
[29]	Aspects of PA counseling (5 A)		Self-developed instrumentValidated via cognitive interviews Pre-tested in pilot study Validity n.r. Paper-pencil survey per mail and online survey Self-report Compensation of €20 for participation	ÄSP-kardio-Study Representative sample of German family physicians A priori defined sample of 13,294	Nationwide; October 2011–March 2012	4074/13,294 (33.9%)	Chi-square test, logistic regressions, missing values n.r.	80.7% assess and 81.3% recommends more PA 87.2% report high or very high competence, 52.3% rated their skills to motivate patients to increase PA as “not good”. Female physicians assess PA more often and provide counseling more often Physicians with a higher proportion of patients at risk for cardiovascular disease provide counseling more often	8/10
[30]	Current state of lifestyle counseling in family practice in the Federal State of Baden-Württemberg. Facilitators and barriers to preventive offers	PA counseling	Self-developed instrument Pre-test Validity n.r. Paper-pencil survey per mail Self-report Expense allowance for participation	General practitioners in Baden-Württemberg randomly selected sample of *n* = 2000	Baden-Württemberg May 2009	260/2000 (13%)	Chi-square test, logistic regressions, missing values n.r.	70.1% assess PA always or often in new patients 54.9% assess and advise PA promotion is offered more often in larger cities and by physicians with high self-reported skill to motivate patients	7/10
[31] (quantitative study part)	State of primary prevention in general practitioners’ practices	PA counseling	Self-developed instrument Pre-test Validity n.r. Paper-pencil survey per mail Self-report	General practitioners in Berlin *n* = 1168	Berlin November 2010–February 2011	474/1168/ (41%)	Descriptive analysis	Approx. 90% of physicians raise the issue of PA if it is indicated	6/10
[32]	Recommendations for preventive offers		Self-developed instrument Pre-test Validity n.r. Paper-pencil survey per mail Self-report	General practitioners in Berlin *n* = 1168	Berlin November 2010–February 2011	474/1168 (41%) 98% of all items were fully answered	Descriptive analysis, Chi-square test	77% of the physicians recommend offers of sports clubs and fitness studios	6/10
**Study Based on Patient Records**									
**Study**	**Primary Research Question**	**Secondary Research Question**	**Data Source**	**Sample**	**Place and Time of the Survey**	**Response Rate**	**Statistics, Dealing Missing Values**	**Main Results**	**Risk of Bias According to Hoy et al.**
[33]	Health promotion, primary and secondary prevention on family practice	PA Counseling	Patient records in 10 family practices in Berlin with more than 1000 patients. Patient at least for three years in the practice	*n* = 500	Berlin 1998 2000	25 practices invited, the first 10 to accept invitation were included	Chi-square test	In 107 (21.4%) patient records, PA counseling was recorded for 63 females vs. 44 males (significantly different) Elderly significantly more often than younger adults	6/10

^1^ PA—physical activity; ^2^ n.r.—not reported; ^3^ BGS 98—National Health Survey Bundesgesundheits survey 1998; ^4^ DEGS 1—First Wave of National Health Survey DEGS; ^5^ OR—odds ratio; ^6^ CI—confidence interval; ^7^ getABI Study—German epidemiological trial on ankle brachial index for elderly patients in family practice to detect peripheral arterial disease; ^8^ EUROPREVIEW Study—cross-sectional study conducted by the European Network for Prevention and Health Promotion in Family Medicine/General Practice; ^9^ ÄSP Study—Physician Survey on Cardiovascular Disease Prevention.

**Table 2:** Qualitative studies.

Study	Primary Research Question	Secondary Research Question	Methods	Sample	Study Place and Time	Data Analysis	Main Results
[34]	Care of arthrosis patients in general practice, views of patients, general practitioners, and practice nurses	Non-drug therapy options for arthrosis patients	Semi-structured interviews with open questions (approx. 45 min.) in the practices	20 general practitioners and 20 practice nurses	Place not reported 2004	Recorded digitally, transcribed literally and analyzed by four different researchers with ATLAS.ti software categorized by four researchers independently	Almost all physicians report regularly mentioning muscle strengthening. Physicians tend to provide general advice. Self-assessed success rate in motivating patients was considered low.
[35]	General practitioners’ and patients’ practices and attitudes regarding overweight encountered during preventive counseling	PA in preventive counseling of overweight patients in general practice	Audiotaped preventive counseling	70 general practitioners were invited, *n* = 12 accepted the invitation, invited *n* = 52 dialogues recorded	Berlin March–September 2007	Recorded digitally, transcribed literally and analyzed by three different researchers with ATLAS.ti softwareQualitative content analysis according to Mayring	PA is the second most common topic in the counseling.
[36]	General practitioners’ and patients’ practices and attitudes regarding overweight encountered during preventive counseling	PA in preventive counseling of overweight patients in general practice	Audiotaped preventive counseling	70 general practitioners were invited, *n* = 12 accepted invitation, invited *n* = 50 dialogues recorded	Berlin Time not reported	Recorded digitally, analysis according to the Roter Interaction Analysis System (RIAS), major themes: cardiovascular risk factors, diet, PA	PA seldom mentioned.
[37]	Obesity management of in general practice from patients’ views	PA as part of the obesity management in general practice	Semi-structured interviews	Overweight and obese patients (*n* = 15; 11 female) out of 52 potential patients	Berlin and Brandenburg	Recorded digitally, transcribed literally using the transcription software “f4” version 3.0.3. Qualitative content analysis according to Mayring Some of the interviews analyzed by two researchers independently	In individual cases a medical history of preferred exercise forms; concrete suggestions for physical activities; Frequent recommendations on the regularity and intensity of PA, with concrete advice on frequency or advice on integrating exercise into everyday life.
[38]	General practitioners’ understanding of prevention, especially as part of the counseling of obese and overweight patients	PA as part of the obesity management in general practice	Semi-structured interviews approx. 54 min in the practices	General practitioners (*n* = 15; 9 female), out of *n* = 78 invited	Berlin and Brandenburg 2006–2007	Recorded digitally, transcribed literally using the transcription software “f4” version 3.0.3. Data analysis ATLAS.ti (Version 5.0) Qualitative content analysis according to Mayring One of the interviews analyzed by two researchers independently	Compared to nutrition, the topic of physical activity receives much less attention. Two physicians recommend patients to participate in cardio sport groups. If at all, physicians typically give general recommendations, recommendations for PA in everyday life, mentioning courses of the health insurance companies or own offers. In one counseling PA is not mentioned at all.
[39]	Exercise on Prescription within the campaign “Berlin on the move” (“Berlin komm(t) auf die Beine”)	Use and relevance of Exercise on Prescription, Benefits and barriers	Semi-structured expert interviews (discussion and pretest) 15–59 min, in th e practices	General practitioners (*n* = 7), who know the campaign “Berlin on the move”, *n* = 244 invited	Berlin September–October 2013	Recorded digitally, transcribed literally using the transcription software “f4” version data analysis according to Meuser and Nagel	None of the physicians use the exercise on prescription in the intended sense; physicians attribute high to very high significance of routine PA counseling in both healthy and diseased patients, the perceived effectiveness of the counseling is either very high or very low.

**Table 3:** Assessment of risk of bias in quantitative studies.

Patient Surveys											
Study	External Validity				Internal Validity						Overall Score
	1	2	3	4	5	6	7	8	9	10	
[15]	low	high	low	high	low	high	high	low	high	low	5/10
[16]	low	high	low	high	low	high	high	low	high	low	5/10
[17]	high	high	high	low	low	high	high	low	high	low	4/10
[18]	high	high	high	high	low	high	high	low	low	low	4/10
[19]	high	low	high	low	low	high	high	low	high	low	5/10
[20]	low	high	low	high	low	high	high	low	high	low	5/10
[21]	high	high	high	high	low	high	high	low	high	low	3/10
[22]	high	high	high	high	low	high	high	low	high	low	3/10
**Physician Surveys**											**Overall Score**
**Study**	**1**	**2**	**3**	**4**	**5**	**6**	**7**	**8**	**9**	**10**	
[23]	high	low	low	high	low	high	high	low	high	low	5/10
[24]	high	low	low	high	low	high	high	low	high	low	5/10
[25]	low	low	low	low	low	high	high	low	low	low	8/10
[26]	low	low	low	low	low	high	high	low	low	low	8/10
[27]	low	low	low	low	low	high	high	low	low	low	8/10
[28]	low	low	low	low	low	high	high	low	low	low	8/10
[29]	low	low	low	low	low	high	high	low	low	low	8/10
[30]	high	low	low	low	low	high	high	low	low	low	7/10
[31] (quantitative part)	high	low	low	high	low	high	high	low	low	low	6/10
[32]	high	low	low	high	low	high	high	low	low	low	6/10

**Table 4:** Assessment of risk of bias in qualitative studies.

Study	Was There a Clear Statement of the Aims of the Research?	Is a Qualitative Methodology Appropriate?	Was the Research Design Appropriate to Address the Aims of the Research?	Was the Recruitment Strategy Appropriate to the Aims of the Research?	Was the Data Collected in a Way that Addressed the Research Issue?	Has the Relationship between Researcher and Participants Been Adequately Considered?	Have Ethical Issues Been Taken into Consideration?	Was the Data Analysis Sufficiently Rigorous?	Is There a Clear Statement of Findings?	How Valuable Is the Research?
[34]	yes	yes	cannot tell	Yes	yes	cannot tell	yes	yes	yes	yes
[35]	yes	yes	cannot tell	cannot tell	yes	cannot tell	cannot tell	yes	yes	yes
[36]	yes	yes	cannot tell	No	yes	cannot tell	yes	yes	cannot tell	yes
[37]	yes	yes	yes	cannot tell	yes	yes	yes	yes	yes	yes
[38]	yes	cannot tell	yes	Yes	yes	yes	yes	yes	yes	yes
[39]	yes	yes	yes	Yes	yes	yes	yes	yes	yes	yes
